# Fructose Intake and Unhealthy Eating Habits Are Associated with MASLD in Pediatric Obesity: A Cross-Sectional Pilot Study

**DOI:** 10.3390/nu17040631

**Published:** 2025-02-10

**Authors:** Maria Felicia Faienza, Jessica Baima, Valentina Cecere, Mariantonietta Monteduro, Ilaria Farella, Rossella Vitale, Valentina Antoniotti, Flavia Urbano, Sabrina Tini, Francesca Romana Lenzi, Flavia Prodam

**Affiliations:** 1 Pediatric Unit, Department of Precision and Regenerative Medicine and Ionian Area, University of Bari “A. Moro”, 70124 Bari, Italy; mariafelicia.faienza@uniba.it; 2 Unit of Endocrinology, Department of Health Sciences, University of Piemonte Orientale, 28100 Novara, Italy; jessica.baima@uniupo.it (J.B.); valentina.antoniotti@uniupo.it (V.A.); sabrina.tini@uniupo.it (S.T.); 3 Giovanni XXIII Pediatric Hospital, University of Bari “A. Moro”, 70124 Bari, Italy; valentinacecere17@gmail.com (V.C.); r.vitale7@studenti.uniba.it (R.V.); flavia.urbano84@gmail.com (F.U.); 4 Department of Diagnostic Imaging, University of Bari “A. Moro”, 70124 Bari, Italy; ma.monteduro@gmail.com; 5 Department of Medicine and Surgery, LUM University, 70010 Casamassima, Italy; ilafarella@yahoo.com; 6 Laboratory of Psychology and Social Processes in Sport, Department of Movement, Human and Health Sciences, University of Rome “Foro Italico”, 00135 Rome, Italy; francescaromana.lenzi@uniroma4.it

**Keywords:** fructose, pediatrics, diet, MASLD, liver, obesity, adolescents

## Abstract

**Background/Objectives**: Fructose consumption in children is increasing, as is the prevalence of metabolic dysfunction-associated steatotic liver disease (MASLD). Despite evidence linking added sugars to metabolic syndrome, fructose’s impact on liver disease in youth remains unclear, especially in pediatrics. Our study aimed to evaluate the role of fructose intake in metabolic and liver dysfunction in a cohort of pre-school children and adolescents with obesity. **Methods**: We recruited 41 children and adolescents with obesity (age range: 2.5–16 years, BMI SDS 2.6 ± 0.5 kg/m^2^). Clinical and biochemical parameters were assessed. Through ultrasound (US), MASLD, hepatorenal index (HRI), subcutaneous adipose tissue (scAT), and visceral adipose tissue (vAT) were assessed. Dietary intake was evaluated using the IDEFICS FFQ and a fructose-specific questionnaire. **Results**: Pubertal subjects had more scAT and vAT, higher insulin resistance, and higher liver fibrosis parameters than those prepubertal. MASLD was detected in 12 subjects, associated with higher scAT and vAT. Pubertal subjects had lower weekly fructose intake than prepubertal subjects (*p* < 0.02). However, they consumed less fructose from fruits (*p* < 0.04) and more from other sugars (*p* < 0.04) than younger children. Patients with MASLD reported higher fructose intake (*p* < 0.01), primarily from fruits (*p* < 0.003), likely due to misreporting, alongside higher consumption of unhealthy food, mainly rich in saturated fats. **Conclusions**: Fructose intake and unhealthy dietary habits were associated with MASLD in pre-school and adolescents with obesity. Advice to pay attention to fructose intake and foods rich in saturated fats is mandatory to decrease both obesity and MASLD. Further high-powered studies in any pediatric age and different geographical areas are needed to better evaluate the MASLD history.

## 1. Introduction

In recent years, high consumption of fructose-rich beverages has been shown to be directly related to the increased incidence of pediatric obesity and its comorbidities [1]. Fructose is a sugar that is more palatable and less capable of inducing satiety; thus, it is found in various processed foods. Free fructose intake is rare, as it is often added as a sweetener in foods and drinks in the form of sucrose (table sugar) or high-fructose corn syrup (HFCS) (55% fructose), commonly used in sugar-sweetened beverages (SSB) [2]. Fructose intake in children and adolescents often exceeds the objective of less than 5% of energy intake from free sugars, i.e., sugars added by the manufacturer, cook, or consumer, plus the sugars present in honey, syrup, or fruit juice.

While fructose intake from fruit is accompanied by a range of nutrients and functional elements such as fiber and antioxidants, sucrose or added sugar in hyperpalatable foods can cause metabolic disorders [3].

Since fructose is less able to promote satiety, it stimulates food consumption and modifies the metabolism of lipids and carbohydrates, thus stimulating the synthesis and accumulation of fat [4,5]. In addition, fructose enhances the action of hepatic pyruvate kinase (L-type), accelerating all glycolysis processes and promoting de novo lipogenesis (DNL) from fructose and glucose. Therefore, glucose and fructose promote the synthesis of subcutaneous and visceral adipose tissues, respectively. Although a certain degree of DNL in adipocytes may be useful to mitigate the high fatty acid deposition in the liver, DNL is currently considered to be a biochemical process underlying some diseases [6].

A high-fructose diet and the widespread commercial use of HFCS are believed to be linked to the increasing prevalence of metabolic syndrome (MetS) globally, which leads to functional impairment of various tissues and organs, resulting in cardiovascular disease (CVD), type 2 diabetes (T2D), metabolic (dysfunction)-associated fatty liver disease (MAFLD), hyperuricemia, gout, and chronic kidney disease (CKD) [7,8]. Numerous studies have shown that high fructose consumption causes fat accumulation, systemic inflammation, oxidative stress, and insulin resistance in many tissues [8]. The dangerous effects of this highly lipogenic sugar have also been observed in infants breastfed by mothers who had ingested this sugar during pregnancy or breastfeeding, who presented metabolic alterations that can last for life [9]. Conversely, replacing fructose with glucose in beverages for 4 weeks improved insulin sensitivity in adipose tissue in young subjects with MAFLD [10].

MASLD (metabolic dysfunction-associated steatotic liver disease) and MAFLD in the pediatric age group represent an advanced categorization of non-alcoholic fatty liver disease (NAFLD), highlighting the intricate relationship between hepatic steatosis and metabolic dysfunction [11,12]. Central or abdominal obesity, a key diagnostic condition for metabolic dysfunction, mainly results from inappropriate or unhealthy dietary habits.

Despite the growing evidence supporting the effects of added sugars on the development of MetS and its related comorbidities, the association between fructose intake and liver diseases remains to be clarified, especially in young people. Recently, we reviewed the observational studies on fructose consumption and MASLD, and almost all the studies confirmed an association between high fructose intake in the diet and a high weight score, as well as an association with MASLD [12]. The studies on MASLD are few compared with those on MetS that demonstrated the association of fructose intake, in particular, with insulin resistance and dyslipidemia [13]. An increased fructose intake seems to have a complex interplay with sedentary behaviors, high consumption of processed food, and socioeconomic and environmental factors in children and adolescents with obesity [12]. However, most studies did not include pre-school children, who could respond differently to metabolic alterations [12,13].

Therefore, this article aimed to investigate the roles of, first, fructose intake, independently of dietary sources, and second, unhealthy dietary habits in metabolic dysfunction and liver steatosis in a cohort of pre-school children and adolescents with obesity.

## 2. Materials and Methods

This cross-sectional study was undertaken at the Unit of Pediatric Endocrinology of University “A. Moro”, Bari, Italy. Anthropometric, biochemical, and instrumental data of pre-school children (age 3–6 years) and adolescents (age 12–16 years) affected by obesity (body mass index (BMI) for age and sex > 97th centile according to WHO 2007) [14], consecutively observed in the period from January 2023 to December 2023, were collected. The BMI cut-off point of >2 SDS was used to define obesity and include patients. Exclusion criteria were genetic causes of obesity and systemic, endocrine, and liver diseases. Written informed consent was obtained from the children’s parents or their legal guardians. All the procedures used were in accordance with the Helsinki Declaration on Human Experimentation guidelines. The study was approved by the Ethics Committee of Policlinico of Bari (protocol number 0002871/12/01/2023).

### 2.1. Measurements

All patients underwent a general clinical examination, and anthropometric measurements were taken (height in cm, weight in kg). BMI was calculated as weight/height^2^ (kg/m^2^) and transformed into a standard deviation score (SDS) based on the Italian BMI reference standards [15]. The pubertal stages were assessed according to the Tanner criteria [16]. Both systolic (SBP) and diastolic blood pressure (DBP) were measured in all patients. Waist circumference was also assessed. And, waist-to-height was calculated (WhR).

### 2.2. Biochemical Assessment

Blood glucose, insulin, glycated hemoglobin A1c (HbA1C), total cholesterol (TC), high- (HDL) and low-density lipoprotein (LDL) cholesterol, triglycerides (TGs), leptin, aspartate aminotransferase (AST), alanine aminotransferase (ALT), gamma-glutamyl transferase (GGT), bilirubin, creatininemia, uric acid, IL1-β, and IL6 were measured after overnight fasting in all subjects. TC, LDL, HDL, and TG values were considered in the normal range if within the 5th and 95th percentiles [17]. An oral glucose tolerance test (OGTT; 1.75 g/kg) was also performed, recording basal levels of blood glucose and insulin after 120 min. Insulin resistance was measured by the homeostasis model assessment (HOMA) index, calculated as insulin (μU/mL) × blood glucose (mmol/L)/22.5 [18].

### 2.3. Hepatic Steatosis, HRI, and Definition of MASLD

All patients underwent liver ultrasound (US) to determine the presence of hepatic steatosis. Currently, US is among the most widely utilized methods for screening hepatic steatosis [19,20]. However, its sensitivity is reduced when fat infiltration levels fall below 30% [21]. The procedure was carried out by a single experienced and trained operator, who was blinded to the patient’s clinical and laboratory data, using a Philips/ATL HDI 5000 scanner (AME, New York, NY, USA). Hepatic steatosis was categorized as follows [20]: grade 1—mild (liver attenuation slightly less than that of the spleen); grade 2—moderate (a more pronounced difference between the liver and spleen, with intrahepatic vessels not seen or with slightly higher attenuation than that of the liver), and grade 3—severe (markedly reduced liver attenuation with a sharp contrast between liver and intrahepatic vessels) [22]. In addition, the hepatorenal index (HRI), a straightforward and dependable ultrasonographic technique, was employed to detect steatosis. PACS software (Agfa IMPAX, edition 6.6.x) was used to calculate variations in echogenicity among different regions of interest (ROIs), assigning grayscale values [18]. An HRI < 1.36 indicates a higher likelihood of the presence of a fatty liver compared to an HRI ≥ 1.36 [23]. The US measurements of intra-abdominal (‘visceral’) and subcutaneous fat were taken using a convex (C5-40R) probe and linear array probe (L12-5) (AME, New York, NY, USA), respectively. US-determined subcutaneous fat was defined as the distance between the skin and the external face of the recto abdominis muscle, and visceral fat was defined as the distance between the internal face of the same muscle and the anterior wall of the aorta.

Metabolic-associated steatotic liver disease (MASLD) in children and adolescents was defined according to the Delphi consensus paper [24]. Specifically, MASLD requires the presence of liver steatosis (in our case, defined through imaging) and at least one specific cardiometabolic risk factor out of the following five: (1) overweight/obesity—i.e., BMI ≥ 85th percentile for age/sex (BMI Z-score ≥ +1) or waist circumference > 95th percentile (values may vary by ethnicity or race); (2) prediabetes/diabetes, evidenced by fasting serum glucose ≥ 100 mg/dL or random serum glucose ≥ 200 mg/dL or 2 h oral glucose tolerance test ≥ 140 mg/dL or HbA1c ≥ 5.7% or an established diagnosis of T2D or specific treatment for T2D; (3) hypertension, indicated by blood pressure (BP) ≥ 130/80 mmHg for age ≥ 13 years; for age < 13 years, BP ≥ 95th percentile or ≥130/80 mmHg (whichever is lower) or the use of antihypertensive treatment; (4) hypertriglyceridemia, defined as triglyceride ≥ 100 mg/dL for age < 10 years or triglyceride ≥ 150 mg/dL for age ≥ 10 years or lipid-lowering treatment; (5) low cholesterol HDL, i.e., HDL ≤ 40 mg/dL, or lipid-lowering treatment.

### 2.4. Basal Metabolism, Dietary Habits, and Fructose Intake

The basal daily energy requirement for each child and adolescent was calculated using Schofield formulas [25]. Dietary habits were evaluated by using the validated Food Frequency Questionnaire from the IDEFICS study [26], 24 h dietary recall, and a structured questionnaire focused on fructose intake, supported by the use of booklets and photos to aid in portion size identification. The history was collected with the involvement of both the children and adolescents and their caregivers. The analysis of the IDEFICS questionnaires determined the total daily caloric intake.

The fructose analysis was conducted using the nutritional software Dietosystem^®^ (Version 18.00.00, DS Medica, Milan, Italy). This software enables the determination of the composition of foods, relying on data from institutional databases (CREA, IEO, USDA) and the nutritional information provided on commercial product labels. For a realistic estimate of the quantity of fructose, the values of interest considered were fructose (when available), sucrose (when available), and simple carbohydrates, quantified for all foods analyzed in the questionnaire. To ensure a more uniform analysis, given the variability in the consumption of these foods, portion sizes were fully standardized (LARN) where gram amounts were not specified in the questionnaire. Assessments were conducted based on weekly consumption.

Fructose was individually extracted for the following foods: fruit, fruit juice, yogurt, sugar, and honey. Fructose from sucrose was obtained by dividing the amount of sucrose in half (one-half glucose, one-half fructose). Sucrose was extracted for the following foods: chocolate (both milk and dark), butter cookies, lemon tea with sugar, fruit juice, ice cream, fruit yogurt, sugar, honey, and jam. Simple carbohydrates include monosaccharides, disaccharides, and oligosaccharides. The reported value for simple carbohydrates is an estimate derived by partitioning the total carbohydrate content into three distinct components.

For sugar-sweetened beverages, individual assessments were conducted to estimate the exact consumption, distinguishing fructose content where known and labeled, and summing fructose, sucrose, and simple carbohydrates based on database or label information. Specifically, sugar-sweetened beverages include fruit juices, canned/bottled teas, sugary sodas, sports drinks, and energy drinks. For the latter three categories, reference values were taken from Coca-Cola (10.5 g of sugar per 100 mL), Gatorade (5.9 g of carbohydrates, of which 3.9 g is sugars per 100 mL), and Red Bull (11 g of sugars per 100 mL).

Since, through the IDEFICS questionnaires, we determined the total daily caloric intake, this allowed for the calculation of the ratio between the daily fructose intake and total caloric intake, providing an estimation of the percentage of daily energy derived from fructose consumption.

### 2.5. Statistical Analysis

Continuous variables are expressed as means ± SD, and categorical variables are reported as numbers and percentages. Statistical significance was defined as *p* < 0.05. We performed statistical comparisons of quantitative data with the nonparametric Mann–Whitney–Wilcoxon test or the Kruskal–Wallis test due to the sample size. For statistical comparisons of dichotomous data, we used the χ2 test. Pearson’s correlation analysis was also performed. All statistical tests were two-sided, with *p* values of <0.05 considered significant. All statistical analyses were performed by using SPSS 27.0 (IBM SPSS Inc., Chicago, IL, USA).

## 3. Results

We recruited 41 subjects (12 males, 29 females) aged 9.7 ± 4.0 years (21 pre-school children and 20 adolescents) affected by obesity (BMI SDS: 2.6 ± 0.5 kg/m^2^; WhR: 0.63 ± 0.12). All pre-school children were prepubertal, and adolescents were pubertal (Tanner stages 3–4). Among them, 29 had normal liver characteristics (70.7%), 8 had mild MASLD (grade 1, MASLD1; 19.5%), and 4 had moderate MASLD (grade 2, MASLD2; 9.8%) without differences in age distribution. However, adolescents had higher BMI SDSs, insulin resistance, elasto-kPa (3.3 ± 0.7 vs. 4.4 ± 1.2, *p* < 0.01), and scAT and vAT than pre-school children, as expected. Males had a prevalence of MASLD marginally higher than females (*p* = 0.08). On the other hand, males were more obese and had a worse metabolic profile than females. Clinical and biochemical characteristics are reported in Table 1 and Table 2.

### 3.1. MASLD and Clinical and Biochemical Characteristics

Subjects with MASLD1 had higher weight SDSs (*p* < 0.02), WhR (*p* < 0.002), total and direct bilirubin (*p* < 0.004), and T-cholesterol (*p* < 0.02) and lower IL1b (*p* < 0.04) than subjects without MASLD. Subjects with MASLD2 had higher WC (*p* < 0.05), WhR (*p* < 0.02), liver enzymes (*p* < 0.001), HOMA-IR (*p* < 0.04), uric acid (*p* < 0.01), and triglycerides (*p* < 0.05) and lower IL1b (*p* < 0.01) and HDL-cholesterol (*p* < 0.002) than subjects without MASLD. Subjects with MASLD together had higher WhR (*p* < 0.001), liver enzymes (*p* < 0.04), total and direct bilirubin (*p* < 0.01), HRI (*p* < 0.03), and scAT (*p* < 0.04) and lower IL1β (*p* < 0.008) than subjects without MAFLD without differences in age distribution (Table 3).

No differences in IL6, leptin, fasting and OGTT glucose, or insulin levels were observed among groups.

### 3.2. MASLD and Fructose Intake

All the groups had similar caloric intake or excess caloric intake with respect to their basal metabolism. Subjects with MASLD1 had a higher intake of fructose (165.6 ± 68.7 vs. 80.4 ± 63.6 g, *p* < 0.004), mainly from fresh fruits (151.2 ± 69.9 vs. 63.9 ± 62.2 g, *p* < 0.001). No differences between MASLD2 and subjects without MASLD were observed due to the high dispersion of fructose intake among the four subjects with MASLD2. However, all subjects with MASLD had a higher intake of fructose (141.4 ± 80.7 vs. 80.4 ± 63.6 g, *p* < 0.01), again mainly from fresh fruits (131.0 ± 73.1 vs. 63.9 ± 62.2 g, *p* < 0.003). Fructose intake from fruits remained higher in patients with MASLD than those without it when related to their weight (2.7 ± 0.5 vs. 1.4 ± 0.3 g/kg, *p* < 0.01).

### 3.3. MASLD and Dietary Habits

Pre-school children ate more sides of raw vegetables (*p* < 0.02), fresh fruits (*p* = 0.05), chocolate cream (*p* < 0.007), and sweet snacks or candies (*p* < 0.05) than adolescents. Adolescents had lower weekly fructose intake than pre-school subjects (*p* < 0.02); however, they consumed less fructose from fruits (*p* < 0.04) and more fructose from other sugars (*p* < 0.04) than younger children.

Subjects with MASLD1 had a higher intake of fresh fruit (*p* < 0.003), and those with MASLD2 had a higher intake of French fries (*p* < 0.01), fried meat and fish (*p* < 0.05), jam (*p* < 0.05), ketchup (*p* < 0.04), white bread (*p* < 0.05), potato chips (*p* < 0.005), and candies (*p* < 0.01) than those without MASLD. Subjects with MASLD together had a higher intake of fresh fruits (*p* < 0.02), jam (*p* < 0.05), potato chips (*p* < 0.05), and candies (*p* < 0.04) and a lower intake of homemade snacks (*p* < 0.05) than subjects without MASLD. When divided according to age, both pre-schooled and adolescents with MASLD ate more fried fish (*p* < 0.02), and the latter also fried potatoes (*p* < 0.02), than those without MASLD.

### 3.4. Correlation Analysis Among Clinical Parameters, Fructose Intake, and Dietary Habits

Uric acid levels, an indirect marker of fructose intake, were associated with sucrose intake (r: 0.343, *p* < 0.05), fructose intake from sucrose (r: 0.343, *p* < 0.05), fructose from SSBs (r: 0.364, *p* < 0.03), the frequency of SSBs (r: 0.351, *p* < 0.04), and chocolate snack intake (r: 0.352; *p* < 0.04), but not with fructose from fruits.

The intake of fried meat was positively associated with the BMI SDS (r: 0.368, *p* < 0.02), triglycerides (r: 0.440, *p* < 0.001), and uric acid (r: 0.478, *p* < 0.005) and negatively correlated with HDL-cholesterol (r: −0.337, *p* < 0.04). BMI SDS was positively associated with candies (r: 0.311, *p* < 0.05), and HOMA-IR with fruit juice (r: 0.329, *p* < 0.04). French fries intake was positively associated with uric acid (r: 0.474, *p* < 0.005). Fructose intake was not associated with clinical and biochemical parameters, apart from MASLD and uric acid (Supplemental Figures S1 and S2).

## 4. Discussion

Pediatric obesity is increasing and is characterized by many comorbidities first described in adulthood. MASLD is also an emerging phenomenon in the pediatric age group [12,27], underlining how its prevention is a health priority. Fructose intake and unhealthy dietary habits have been identified as key factors in the development of MASLD [8,12,28]. In a population of pre-school and adolescents with pediatric obesity, we identified a high prevalence of MASLD. The US characteristics of MASLD are directly associated with the burden of clinical and metabolic characteristics of obesity as well as with worse dietary habits.

### 4.1. MASLD Prevalence Between Groups

First, we identified a prevalence of about 30% for MASLD, similar between pre-school children and adolescents with obesity. In our cohort, the prevalence seems similar and independent of age and, indeed, puberty, although insulin resistance is higher in adolescents, as expected. A recent review by our group summarized pediatric studies on MAFLD/MASLD that included a total of 1485 children and adolescents, mainly Caucasian, with overweight or obesity [12]. The reported prevalence in the retrieved papers and other papers varied from 8% to 60% of pediatric subjects with overweight or obesity [29,30,31,32,33]. The differences are related to how MASLD/MAFLD was diagnosed, the criteria for the recruitment of the populations, the country’s lifestyle habits, food insecurity, and the year of publication. Adolescents were the most numerous cohorts in any studies, whereas pre-school children were few and, when present, aged 5–6 years old [12,28,29,32,33,34]. Indeed, data on MASLD in very young children with obesity are still limited, but it seems similar in children and adolescents [29]. Furthermore, we observed a slightly higher, although not significant, prevalence in males than in females. This finding is controversial since some studies reported gender differences in pediatric subjects with obesity but not in the general population [29,33]. Furthermore, the onset and progression of puberty with high estradiol levels in males and changes in body composition seem to be associated with the differences in prevalence between genders [35]. This could be due to several reasons, including ethnicity, publication bias (absence of stratification for gender), and unreported lifestyle habits (such as alcohol intake in adolescents). On the other hand, in our population, males presented a worse metabolic profile than females, with a hypothetical effect on MASLD prevalence. We did not observe metabolic differences between pre-school children and adolescents with MASLD, and this point could be due to the criteria through which MASLD is diagnosed [11]. However, visceral obesity and metabolic alterations, particularly high uric acid and insulin resistance, increased progressively with the grade of MASLD. These findings, in line with other recent reports, suggest that the excess adipose tissue and the burden of comorbidities are drivers or epiphenomena of MASLD, independently of age or puberty [12,29,36,37]. These results support the novel definition of the condition, highlighting its metabolic etiology.

### 4.2. MASLD and Metabolic and Biochemical Parameters

Interestingly, liver enzymes were higher in subjects with MASLD. However, patients who did not meet the diagnostic criteria for MASLD also had high liver enzymes, particularly ALT levels. A recent report evaluates ALT levels in more than 120,000 children, reporting high ALT levels in 7.2% and 16.8% of those with overweight and obesity, respectively. The increased ALT levels were associated with a worse cardiometabolic profile [38]. Similar results regarding correlations of ALT with components of MetS have been reported in several other cohorts, although less numerous [39,40]. All the authors suggested that an increase in ALT levels is associated not only with fatty liver disease but also with general metabolic dysfunction. High ALT levels could entail a progressive deterioration in the parameters included in MetS. Indeed, in our patients without MASLD, high liver enzymes could suggest a preclinical stage of MASLD associated with an early subtle metabolic derangement.

Different from several reports [41], we failed to identify differences in circulating IL6 levels. This could be due to the fact that all the subjects presented comorbidities, insulin resistance, and severe obesity, suggesting that chronic inflammation was present in all of them. On the other hand, surprisingly, IL1β levels were indirectly associated with the MASLD grade, independently of age. IL1β seems to be a marker for the advancement of MAFLD [42]. IL1β is mostly related to the late stages of inflammation due to the recruitment of macrophages and could activate Kupffer cells, exacerbating the inflammatory response and causing liver injury and hepatic fibrosis. However, since IL1β is a crucial player in initiating and maintaining tissue damage, it is possible that, in children and adolescents with obesity, its levels are higher systemically, and when liver damage begins to progress, IL1β is more present locally in the target tissues [42,43,44].

### 4.3. MASLD and Fructose Intake

Lifestyle changes remain the cornerstone in the treatment of obesity. Improving dietary habits is crucial for the long-term prevention of excess adipose tissue but also for comorbidities [45,46]. Several papers have identified sugars, in addition to other unhealthy foods, as critical players in the obesity epidemic. Chronic over-nutrition favors hepatic steatosis and fibrosis in both childhood and adulthood. A long-term high-calorie diet mostly rich in saturated fatty acids (SFAs) leads to increased hepatic gluconeogenesis, insulin resistance, and hepatic lipid accumulation of almost 50% [47,48]. Furthermore, recent evidence underlines a correlation between fructose intake and MASLD [12,48]. Several findings indicated a daily average of 132 kcal from HFCS for all Americans aged > 2 years, and the top 20% of consumers of calories reached 316 kcal [49]. Free sugar intake in childhood is mostly accounted for by sweets (34%) and fruit juices (22%), but also SSBs, particularly in adolescents, whereas data on young children are less precise [12,50].

In our population with obesity, we recorded the main unhealthy habits associated with the nutrition transition to a Western diet. Regarding our primary outcome, we recorded a higher intake of sugary foods in our subjects with obesity and MASLD, in line with reports in adulthood [48]. We observed an association between MASLD, its US grade, and fructose intake, as reported by the recent literature [12,32,35,36]. However, surprisingly, we failed to observe a role for fructose or other sugars from SSBs, but we recorded an association with fructose derived from fruits. Since most of our subjects reported more than three servings of fruits daily, and some more than six servings, our findings could be linked to an excess of these. The misreporting of fruits instead of fruit juice or SSBs could not be excluded. Recently, the European IDEFICS/I. Family and the Amsterdam Born Children and their Development Cohort Consortia showed negative associations between sugar-sweetened foods and BMI starting from 11 years old and no associations in children younger than 6 years. These results were unexpected and surprising, since sugars, mainly in SSBs and processed foods, are well-established risk factors for obesity. The authors justified these findings in relation to the fact that foods considered unhealthy may be susceptible to misreporting due to socially desirable adequate behavior [51]. Moreover, since we used the same food frequency questionnaire, we cannot exclude that the questions are not precise enough to detect fructose from sources different from fruits. Furthermore, we used a second semi-quantitative questionnaire to capture the amount of fructose in the diet, but several foods do not report it on labels or in institutional databases. On the other hand, too many servings of fruits (>4 portions) are known to exacerbate steatosis, dyslipidemia, and glycemic control in adults with MASLD [52]. The suggested servings of fruits and vegetables together are five a day, or 250–400 g per day in relation to age, according to the WHO guidelines [53]. Avoiding surpassing recommended servings or using fruits as a substitute for other foods in cases of overeating could be a partial health solution, at least in the short term, since it is possible that the negative effect of fruits is not related to the fructose inside but is an indirect marker of caloric overload. On the other hand, we observed positive associations among uric acid levels, a well-known marker of fructose/sucrose intake, and fructose intake from sucrose, fructose from SSBs, and the frequency of weekly intake of SSBs and chocolate snacks, but not with fructose from fruits. These findings support a misreporting bias from patients and/or caregivers and are likely more due to fructose from processed food. Unfortunately, the questionnaires we used were not designed and validated to classify the amount of unprocessed, minimally processed, and ultra-processed foods to obtain more information on this point.

### 4.4. MASLD, Metabolic Profile, and Other Unhealthy Dietary Habits

As previously introduced, we observed several unhealthy habits in our population. Although our cohort lives in the Mediterranean Sea area, Southern Italy children and adolescents, similarly to those living in Northern Italy, more often have an average or poor adherence to the Mediterranean diet [54,55,56,57]. Furthermore, the Italian Nutrition & Health Survey reported that ultra-processed food consumption accounts for one-quarter of the total energy in children and adolescents [58]. In detail, we observed that, apart from sugary foods, the intake of fried foods, rich in saturated fats, is high in our subjects with obesity and MASLD, again in line with reports in adulthood [48]. No main differences were shown according to age, although adolescents with MASLD consumed more fried chips than those who were younger, likely due to self-decision on what to eat out of the home with their peers [59]. Interestingly, subjects with obesity without MASLD had a higher intake of homemade snacks, suggesting that policies to reduce the daily amounts of processed foods and industrial snacks are a cornerstone in the prevention of both obesity and its comorbidities [46,60].

Furthermore, all these unhealthy habits were associated in our population with a worse metabolic profile, as expected. Fried foods, particularly meat, were directly associated with a higher BMI SDS and a worse lipid profile. Saturated fats and molecules produced during frying, including trans fats, are factors well known to be causative for atherosclerosis. On the other hand, they are also linked to an increased BMI in both children and adults, as highlighted by the recent WHO guidelines [61,62]. Several studies conducted in the pediatric age group demonstrated that reducing them improved BMI and lipid profiles [63,64]. Furthermore, an increased meat intake has been associated with overweight and obesity in children, adolescents, and adults [65,66]. Moreover, we confirmed the association between candy and fruit juice intake with insulin resistance [67,68].

We also observed an association with foods rich in saturated fats and uric acid. These findings could have several explanations. First, saturated fats promote lipogenesis, and adipose tissue is also able to produce uric acid [69]. Second, these dietary habits could hide the coupled intake of foods rich in saturated fats and fructose. Since high fructose exacerbates the effects of a high-fat diet alone by several mechanisms, as recently demonstrated, in particular, in animal models [13], these results deserve further attention.

### 4.5. Limitations

This study has several limitations. First, our population is limited. However, we included many pre-school children and young children, in contrast to most other studies focused on adolescents. Data on subgroups of patients with MASLD could be partly inconsistent due to the sample size. However, this is a pilot study that also offers a statistical basis for higher-powered studies, particularly in the population younger than 6 years. Second, we did not perform liver biopsies. On the other hand, we defined MASLD with a well-defined radiological protocol. Third, the food frequency questionnaire seems not to be precise enough to capture the total amount of fructose in the diet. However, we used a semi-quantitative dedicated questionnaire and the IDEFICS questionnaire, which is one of the most widely used questionnaires for dietary research in the pediatric age group. These observations strengthen the importance of clarity in food labels and of having more information on the composition of processed foods in databases and software used for detecting dietary habits. Fourth, we did not have data on physical activity. Our adolescents performed only physical activity at school, whereas no validated questionnaire on pre-school children is available to have strong data to compare the subpopulations. Further strategies to collect this information in young children are advisable for further studies.

### 4.6. Perspectives

This study opens further research directions. First, studies including large sample sizes, including any pediatric age and puberty stage, are needed to understand the history of MASLD. New questionnaires focused on fructose intake and ultra-processed food consumption for children and adolescents should be designed. Markers of fructose intake, apart from uric acid levels, through omics signatures could help in clarifying the natural history of MASLD in pediatric obesity and identifying new mechanisms or clusters of patients at the highest risk. The inclusion of different geographical areas to detect the impact of lifestyle habits in different environments is needed. Social and environmental determinants and their role in the research on dietary habits are warranted.

## 5. Conclusions

In conclusion, MASLD was present in both pre-school children and adolescents with obesity and related to unhealthy dietary habits. Advice to pay attention to foods rich in fructose and saturated fats is mandatory to decrease both pediatric obesity and MASLD. Educational programs with simple suggestions for children, parents, and caregivers at schools should be encouraged.

## Figures and Tables

**Table 1:** Characteristics of the population at baseline.

Variables	Pre-School 3–6 yrs	Adolescents 12–16 yrs	Total Population
Gender (M/F)	M: 2 F: 19	M: 10 F: 10	M: 12 F: 29
Age (yrs)	5.6 ± 1.4	13.4 ± 1.6 ***	9.0 ± 4.0
Weight (Kg)	38.8 ± 10.5	85.7 ± 16.2 ***	61.7 ± 27.3
Height SDS	0.7 ± 1.0	0.7 ± 1.0	0.7 ± 1.0
BMI (kg/m^2^)	25.9 ± 4.1	32.2 ± 5.4 **	28.9 ± 5.7
BMI-SDS	2.4 ± 0.6	2.9 ± 0.4 **	2.7 ± 0.6
WC (cm)	80.2 ± 10.5	102.8 ± 11.5 ***	91.5 ± 15.7
WhR	0.63 ± 0.16	0.63 ± 0.07	0.63 ± 0.12
SBP (mmHg)	101.5 ±13.6	118.2 ± 12.6 **	109.9 ± 15.5
DBP (mmHg)	64.7 ± 9.8	71.7 ± 10.7 *	68.2 ± 10.7
Glucose T0’ (mg/dL)	82.3 ± 9.2	85.3 ± 6.8	83.8 ± 8.1
Glucose T120’ (mg/dL)	106.5 ± 26.7	101.6 ± 24.4	104.1 ± 25.4
Insulin T0’ (μU/mL)	16.2 ± 16.7	26.1 ± 19.9 **	21.1 ± 18.8
Insulin T120’ (μU/mL)	83.5 ± 80.6	142.8 ± 161.8 *	114.7 ± 73.0
HbA1c (mmol/mol)	34.9 ± 2.9	33.4 ± 5.4	34.2 ± 4.4
HOMA-Index	3.3 ± 3.4	5.7 ± 4.8 ***	4.5 ± 4.3
Total-c (mg/dL)	141.6 ± 24.2	138.8 ± 43.0	140.3 ± 34.2
HDL-c (mg/dL)	46.5 ± 8.5	40.3 ± 8.8 *	43.5 ± 9.1
LDL-c (mg/dL)	78.7 ± 21.7	89.1 ± 28.4	83.7 ± 25.4
TG (mg/dL)	84.9 ± 52.1	89.9 ± 52.9	87.3 ± 51.9
AST (U/L)	31.6 ± 19.3	25.1 ± 9.6	28.4 ± 15.5
ALT (U/L)	49.2 ± 77.5	32.8 ± 19.0 *	41.2 ± 57.0
GGT (U/L)	33.7 ± 45.7	24.0 ± 14.8 *	28.7 ± 33.5
Leptin (ng/mL)	14.4 ± 7.4	20.8 ± 10.2	17.9 ± 9.5
IL-6 (pg/mL)	4.8 ± 3.8	14.8 ± 26.9	9.8 ± 19.6
IL-1β (pg/mL)	12.5 ± 8.6	12.6 ± 9.9	12.6 ± 9.2
HRI	1.49 ± 0.44	1.41 ± 0.25	1.46 ± 0.37
vAT (cm)	0.8 ± 0.4	1.5 ± 0.7 **	1.1 ± 0.6
scAT (cm)	2.0 ± 0.7	3.2 ± 0.9 **	2.5 ± 0.9

Legend. Data are represented as mean ± standard deviation. BMI: body mass index; BMI-SDS: body mass index—standard deviation score; WC: waist circumference; WhR: waist-to-height ratio; SBP: systolic blood pressure; DBP: diastolic blood pressure; HbA1c: glycated hemoglobin; HOMA-Index: homeostasis model assessment of insulin resistance; Total-c: total cholesterol; LDL-c: low-density lipoprotein cholesterol; HDL-c: high-density lipoprotein cholesterol; TG: triglyceride; AST: Aspartate Aminotransferase; ALT: Alanine Aminotransferase; GGT: gamma-glutamyl transferase; IL-6: Interleukin-6; IL-1β: Interleukin-1beta. * *p* < 0.05; ** *p* < 0.01; *** *p* < 0.0001 (comparison between pre-school children and adolescents).

**Table 2:** Characteristics of the population divided by gender.

Variables	Males	Females
Gender (M/F)	N: 12	N: 29
Age (yrs)	12.4 ± 3.1	8.5 ± 3.8 **
Weight (Kg)	83.6 ± 22.3	52.6 ± 23.9 **
Height SDS	0.8 ± 1.1	0.7 ± 1.0
BMI (kg/m^2^)	32.7 ± 6.0	27.5 ± 4.8 **
BMI-SDS	2.9 ± 0.5	2.5 ± 0.5 *
WC (cm)	107.5 ± 10.5	84.6 ± 12.2 ***
WhR	0.68 ± 0.08	0.61 ± 0.13 *
SBP (mmHg)	121.2 ± 11.3	105.0 ± 14.5 **
DBP (mmHg)	72.5 ± 12.1	66.4 ± 9.7
Glucose T0’ (mg/dL)	87.9 ± 5.3	82.1 ± 8.5 *
Glucose T120’ (mg/dL)	106.7 ± 24.8	102.9 ± 25.9
Insulin T0’ (μU/mL)	28.6 ± 23.6	17.9 ± 15.8 *
Insulin T120’ (μU/mL)	179.5± 198.6	84.8 ± 73.1 *
HOMA-Index	6.4 ± 5.8	3.7 ± 3.2 *
Total-c (mg/dL)	135.8 ± 23.1	142.1 ± 38.1
HDL-c (mg/dL)	40.0 ± 10.5	44.9 ± 8.2 *
LDL-c (mg/dL)	82.1 ± 20.6	84.4 ± 27.5
TG (mg/dL)	88.2 ± 55.1	86.9 ± 51.5
AST (U/L)	30.8 ± 14.0	27.5 ± 16.3
ALT (U/L)	43.8 ± 28.7	40.1 ± 65.6
GGT (U/L)	30.3 ± 17.4	28.0 ± 38.9
Leptin (ng/mL)	21.5 ± 13.2	16.4 ± 7.3
IL-6 (pg/mL)	11.5 ± 10.6	9.1 ± 22.5 *
IL-1β (pg/mL)	8.6 ± 7.5	14.3 ± 9.4 *
HRI	1.53 ± 0.34	1.44 ± 0.38
vAT (cm)	1.6 ± 0.5	0.9 ± 0.5 **
scAT (cm)	3.4 ± 0.8	2.2 ± 0.8 **

Legend. Data are represented as mean ± standard deviation. BMI: body mass index; BMI-SDS: body mass index—standard deviation score; WC: waist circumference; WhR: waist-to-height ratio; SBP: systolic blood pressure; DBP: diastolic blood pressure; HbA1c: glycated hemoglobin; HOMA-Index: homeostasis model assessment of insulin resistance; Total-c: total cholesterol; LDL-c: low-density lipoprotein cholesterol; HDL-c: high-density lipoprotein cholesterol; TG: triglyceride; AST: aspartate aminotransferase; ALT: alanine aminotransferase; GGT: gamma-glutamyl transferase; IL-6: Interleukin-6; IL-1β: Interleukin-1beta. * *p* < 0.05; ** *p* < 0.01; *** *p* < 0.0001 (comparison between males and females).

**Table 3:** Characteristics of the population divided by MASLD grade.

Variables	NO MASLD	MASLD1	MASLD2	MASLD
Gender (M/F)	N: 29	N: 8	N: 4	N: 12
Age (yrs)	10.2 ± 4.1	7.6 ± 3.6	10.6 ± 4.2	8.6 ± 3.9
Weight (kg)	61.4 ± 26.5	52.8 ± 21.9	81.6 ± 38.9	62.4 ± 30.3
Height (cm)	143.4 ± 22.7	132.2 ± 24.0	150.0 ± 21.4	138.1 ± 23.9
BMI (kg/m^2^)	28.2 ± 5.1	28.9 ± 4.2	34.7 ± 10.0	30.8 ± 6.8
BMI-SDS	2.6 ± 0.6	2.8 ± 0.6	3.0 ± 0.7	2.9 ± 0.6
WC (cm)	89.2 ± 14.9	92.2 ± 14.4	106.3 ± 20.1	96.9 ± 17.0
WhR	0.60 ± 0.13	0.70 ± 0.07	0.71 ± 0.08	0.70 ± 0.07
PAS (mmHg)	110.4 ± 16.3	108.1 ± 12.2	110.0 ± 18.7	108.8 ± 13.8
PAD (mmHg)	68.6 ± 10.4	63.1 ± 8.8	76.3 ±13.8	67.5 ± 12.0
Glucose T0’ (mg/dL)	84.2 ± 9.1	81.9 ± 6.4	84.8 ± 2.2	82.8 ± 5.4
Glucose T120’ (mg/dL)	105.5 ± 25.5	101.9 ± 30.8	99.0 ± 15.1	100.9 ± 25.8
Insulin T0’ (μU/mL)	20.7 ± 20.5	16.8 ± 11.8	32.2 ± 16.7	21.9 ± 14.9
Insulin T120’ (μU/mL)	127.2 ± 154.0	90.8 ± 68.8	81.6 ± 28.3	87.7 ± 57.0
HOMA-Index	4.4 ± 4.7	3.5 ± 2.6	6.7 ± 3.5	4.5 ± 3.2
Total-c (mg/dL)	135.5 ± 36.5	158.0 ± 22.4	139.5 ± 30.6	151.8 ± 25.7
HDL-C (mg/dL)	43.8 ± 8.4	48.1 ± 9.0	31.5 ± 1.3	42.6 ± 10.9
LDL-C (mg/dL)	82.0 ± 25.5	93.4 ± 25.5	76.8 ± 26.1	87.8 ± 25.9
TG (mg/dL)	79.3 ± 41.1	82.4 ± 39.0	155.3 ± 98.6	106.7 ± 70.1
AST (U/L)	27.2 ± 16.4	22.9 ± 1.6	48.3 ± 8.8	31.3 ± 13.4
ALT (U/L)	39.0 ± 65.6	28.6 ± 8.3	82.5 ± 13.8	46.6 ± 28.3
GGT (U/L)	28.1 ± 38.2	22.9 ± 10.2	43.3 ± 22.3	30.3 ± 17.8
Leptin (ng/mL)	16.2 ± 7.3	21.4 ± 9.4	24.2 ± 19.0	22.5 ± 13.1
IL-6 (pg/mL)	11.0 ± 23.3	6.5 ± 4.1	8.1 ± 5.7	7.1 ± 4.5
IL-1β (pg/mL)	14.6 ± 8.9	9.3 ± 9.8	5.7 ± 5.6	8.1 ± 8.5
HRI	1.4 ± 0.4	1.6 ± 0.4	1.6 ± 0.3	1.6 ± 0.4
vAT (cm)	1.1 ± 0.7	1.2 ± 0.7	1.2 ± 0.6	1.1 ± 0.6
scAT (cm)	2.4 ± 0.9	3.0 ± 1.2	3.1 ± 1.1	3.0 ± 1.1

Legend. Data are represented as mean ± standard deviation. BMI: body mass index; BMI-SDS: body mass index—standard deviation score; WC: waist circumference; WhR: waist-to-height ratio; SBP: systolic blood pressure; DBP: diastolic blood pressure; HbA1c: glycated hemoglobin; HOMA-Index: Homeostasis model assessment of insulin resistance; total-c: total cholesterol; LDL-c: low-density lipoprotein cholesterol; HDL-c: high-density lipoprotein cholesterol; TG: triglyceride; AST: aspartate aminotransferase; ALT: alanine aminotransferase; GGT: gamma-glutamyl transferase; IL-6: Interleukin-6; IL-1β: Interleukin-1beta; HRI: hepatorenal index; scAT: subcutaneous adipose tissue; vAT: visceral adipose tissue. Significance is reported in the main text. Table reported raw data. Significances in the text were not modified by correction for BMISDS or age.

## Data Availability

The data presented in this study are available on request from the corresponding author due to ethical reasons.

## Supplementary Materials

The following supporting information can be downloaded at: https://www.mdpi.com/article/10.3390/nu17040631/s1, Figure S1: Correlation between uric acid and fructose intake from dietary habits; Figure S2: Correlation between clinical parameters and fructose intake from dietary habits.

## Abbreviations

The following abbreviations are used in this manuscript:

HFCS	High-fructose corn syrup
SSB	Sugar-sweetened beverages
Mets	Metabolic syndrome
CVD	Cardiovascular disease
T2D	Type 2 diabetes
MAFLD	Metabolic (dysfunction)-associated fatty liver disease
CKD	Chronic kidney disease
MASLD	Metabolic dysfunction-associated steatotic liver disease
NAFLD	Non-alcoholic fatty liver disease
BMI	Body mass index
SDS	Standard deviation score
SBP	Systolic blood pressure
DBP	Diastolic blood pressure
WhR	Waist-to-height ratio
HbA1c	Glycated hemoglobin A1c
TC	Total cholesterol
HDL	High-density lipoprotein cholesterol
LDL	Low-density lipoprotein cholesterol
TG	Triglycerides
AST	Aspartate aminotransferase
ALT	Alanine aminotransferase
GGT	Gamma-glutamyl transferase
IL1-β	Interleukin 1-β
IL6	Interleukin 6
OGTT	Oral glucose tolerance test
HOMA	Homeostasis model assessment
US	Liver ultrasound
HRI	Hepatorenal index
ROIs	Regions of interest
SFAs	Saturated fatty acids
