# Supplementary material for: Fructose Intake and Unhealthy Eating Habits Are Associated with MASLD in Pediatric Obesity: A Cross-Sectional Pilot Study
Source: Nutrients. 2025 Feb 10;17(4):631. doi: 10.3390/nu17040631 (PMC11858415; doi:10.3390/nu17040631)
Supplement: Supplementary file 1 [file nutrients-17-00631-s001.zip › Supplemental Figure 2.pdf]

Supplemental Figure 2 - Correlation between clinical parameters and fructose intake from dietary habits

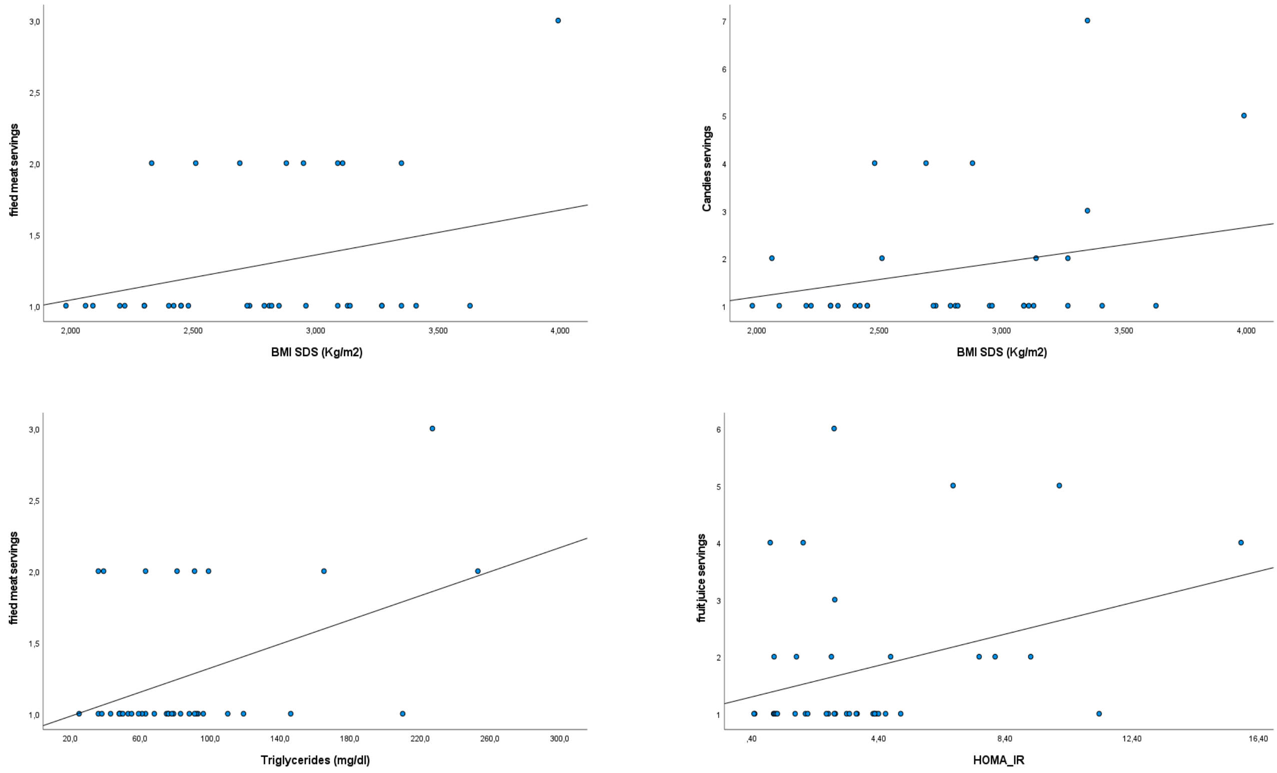

[1]Legend. Data were represented as correlation plots. as mean ± standard deviation. BMI: Body mass index; BMI-SDS: Body mass index – Standard Deviation Score; HOMA-IR: Homeostasis model assessment of insulin resistance.
